# Supplementary material for: Hybrid Unilamellar Vesicles of Phospholipids and Block Copolymers with Crystalline Domains
Source: Polymers (Basel). 2020 May 29;12(6):1232. doi: 10.3390/polym12061232 (PMC7362021; doi:10.3390/polym12061232)
Supplement: Supplementary file 1 [file polymers-12-01232-s001.zip › SI/Supporting Information_may25_2020.docx]

Supporting Information

Hybrid Unilamellar Vesicles of Phospholipids and Block Copolymers with Crystalline Domains

Yoo Kyung Go, Nurila Kambar, and Cecilia Leal*

Department of Materials Science and Engineering, University of Illinois at Urbana−Champaign, Urbana, Illinois 61801, USA; [go6@illinois.edu](mailto:go6@illinois.edu) (Y. K. G.); [nkambar2@illinois.edu](mailto:nkambar2@illinois.edu) (N. K.)

***** Correspondence: [cecilial@illinois.edu](mailto:cecilial@illinois.edu)

**Keywords:**  giant hybrid vesicles; phospholipids; diblock copolymer; semi-crystalline polymer; phase-separation;


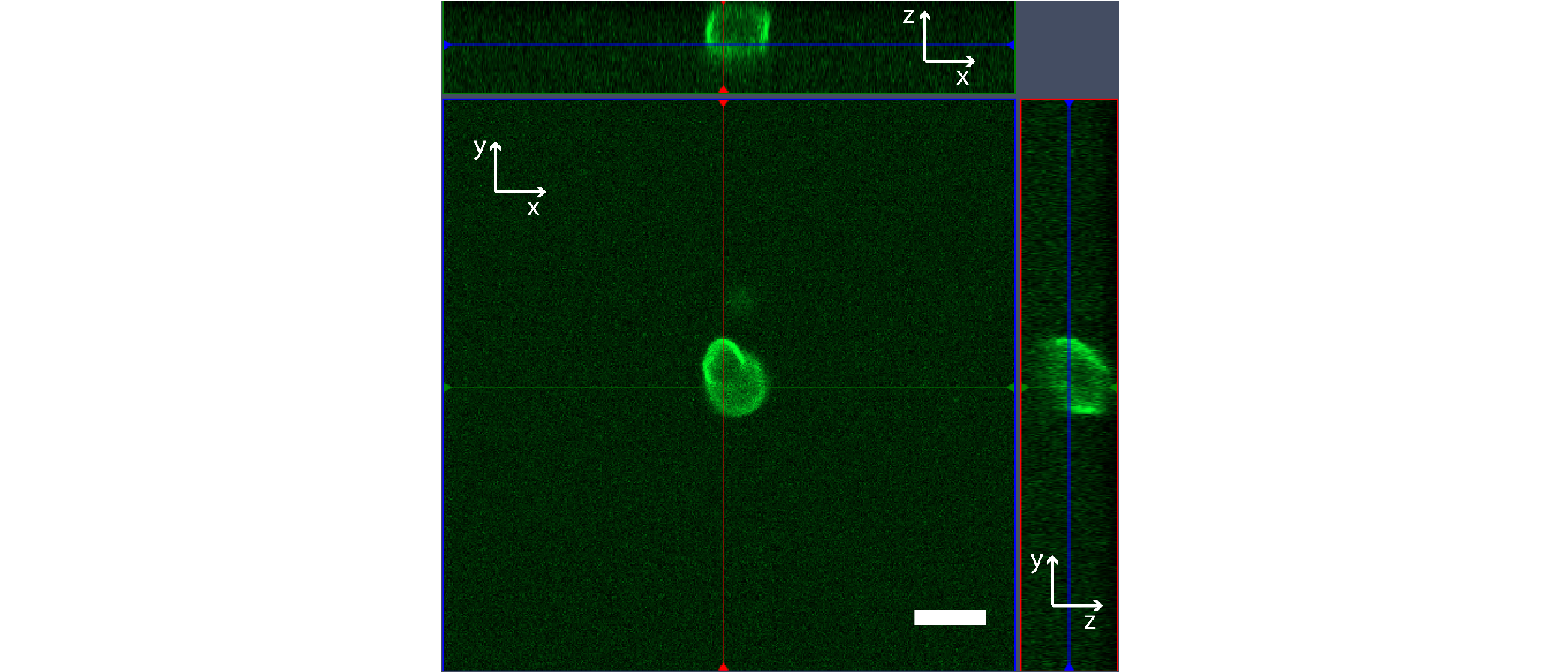


**Figure S1.** 2D cross-sectional CLSM images of the mPEG-*b*-PCL BCP-GUV in **Figure 3b** at x-y(*middle*), x-z(*top*) and y-z(*right*) planes (Scale bar: 10 μm). (mPEG-*b*-PCL)-FITC was used for fluorescent labelling. RL-FITC fluorescence in green (excitation laser at 488 nm / detection wavelength 400-571 nm). The GUV encapsulated 100 mM sucrose buffer and was suspended in iso-osmolar solution of 100 mM glucose to lower the mobility during microscopic observation. All images were obtained on different focal planes by z-stacking on Plan-Apochromat 20×/0.8 M27 objective lens. Z-stack thickness: 0.5 μm. This Figure S1. is reproduced from the Z-stack images by software Zenblue (Carl Zeiss Microimaging GmbH, Germany).

﻿


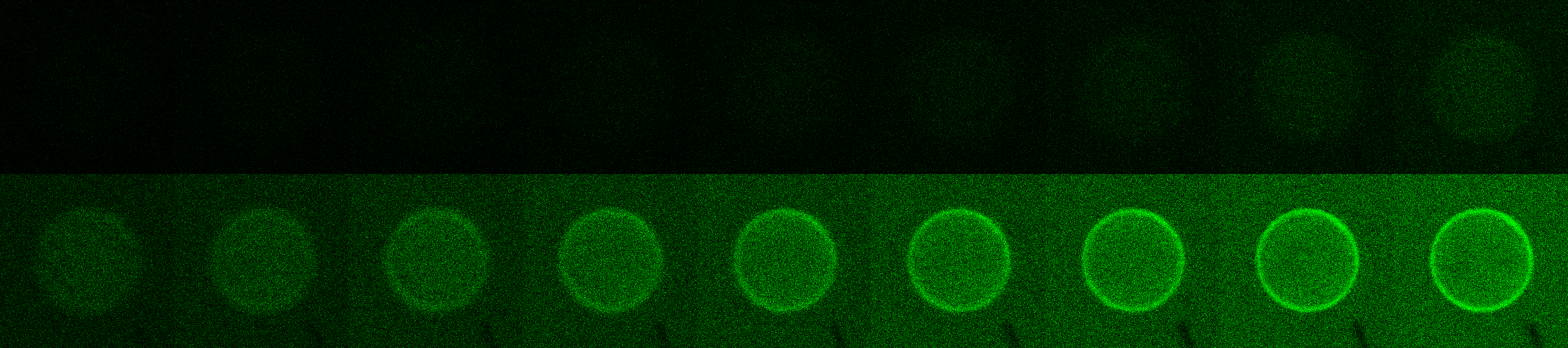


(a)


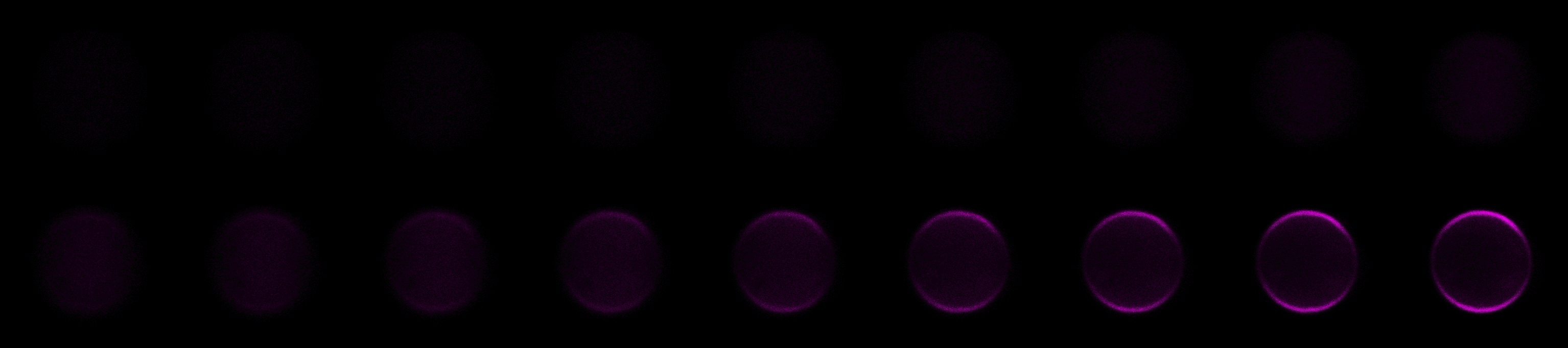


(b)

**Figure S2.** Height-resolved Z-stack CLSM images of the upper hemisphere of the DPPC - mPEG-*b*-PCL GHUV in **Figure 4** (80 mol% DPPC with respect to mPEG-*b*-PCL). (mPEG-*b*-PCL)-FITC and 16:0 Liss Rhod PE were used to tag polymer domains and lipid domains, respectively. Two separate channels were used; (a) RL-FITC fluorescence in green (excitation laser at 488 nm / detection wavelength 400-544 nm) (b) RL-Rhod B fluorescence in magenta (excitation laser at 561 nm / detection wavelength 558-700 nm) Arrow direction is towards the hemisphere plane. Z-stack thickness : 1 μm. Images were obtained on EC Plan-Neofluar 10×/0.3 Ph 1 objective lens. The internal and external medium of the vesicles is MilliQ water.


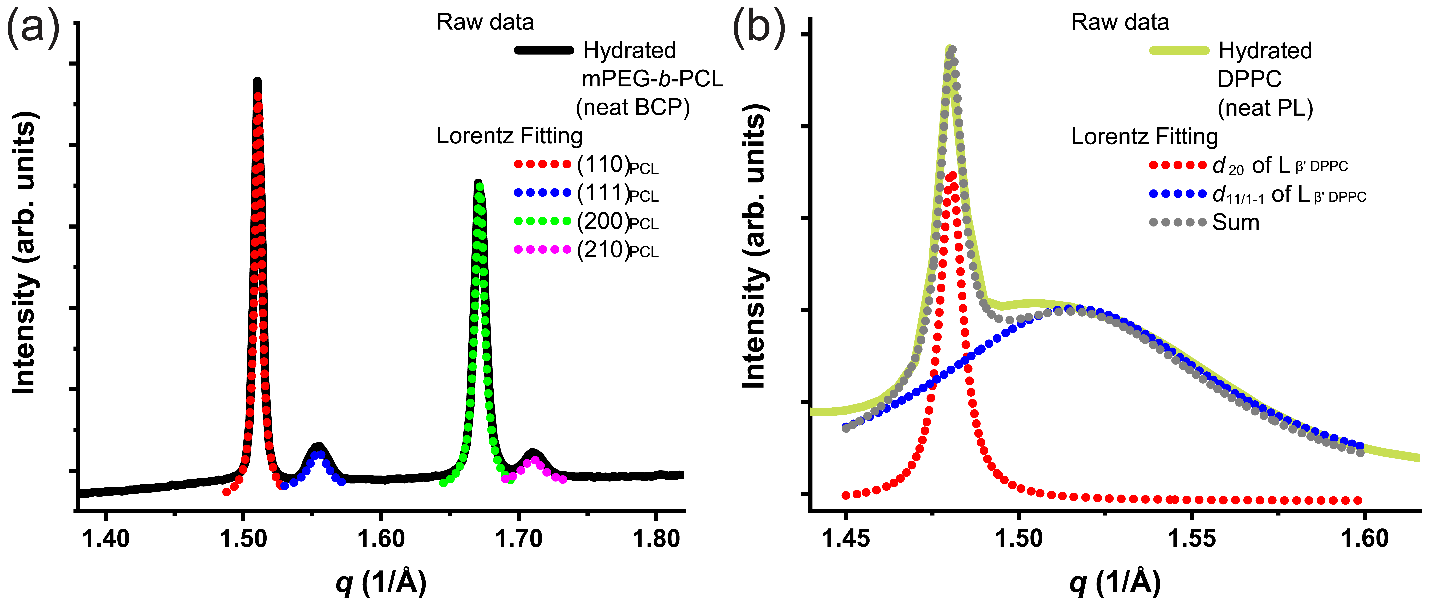


**Figure S3.** Lorentzian fitting of 1D Wide Angle X-ray Scattering profiles in Figure 5b. (a) Hydrated mPEG-*b*-PCL or neat BCP. The diffraction of planes (110), (111), (200), and (210) of a PCL orthorhombic unit cell appear at four peaks at *q* = 1.51, 1.55, 1.67, and 1.71 $Å$^-1^, respectively. (b) Hydrated DPPC or neat PL. The diffraction peaks at *q*= 1.48 and 1.52 $Å$^-1^ originates from the packing of DPPC alkyl chains in an pseudo-hexagonal unit cell (PL gel phase) with *a* = 8.46 and *b*= 4.71$Å$. The information of the fitting curves in this figure is **Table S1** below. This fitting was performed in software Origin(Pro), Version 2020 (OriginLab Corporation, Northampton, MA, USA.).

**Table S1**.

Parameters of the Lorentz Fitting used for the fitted curves in 1D Wide Angle X-ray Scattering profiles in **Figure 5b** and **Figure S3** of (a) hydrated mPEG-*b*-PCL or neat BCP and (b) hydrated DPPC or neat PL. Equation: y = y0 + (2*A/pi)*(w/(4*(x-xc)^2 + w^2)), where y0 is the offset, xc is the center, w is the width, and A is the area of the curve. Every peak fitted here displays a good-fit to a Lorentz function.

| (a) hydrated mPEG-*b*-PCL (BCP) | Peak1  ((110) of orthorhombic PCL) | Peak2  ((111) of orthorhombic PCL) |
| --- | --- | --- |
| y0 (arb. units) | 0.06473 ± 0.00434 | 0.07594 ± 0.00155 |
| xc ($Å$^-1^) | 1.51077 ± 7.79044E-5 | 1.55439 ± 2.35605E-4 |
| w ($Å$^-1^) | 0.00624 ± 2.64684E-4 | 0.01785 ± 0.00135 |
| A (arb. units) | 0.00486 ± 1.82627E-4 | 0.00129 ± 1.18561E-4 |
| Reduced Chi-Sqr | 1.19108E-4 | 3.66764E-6 |
| R-Square (COD) | 0.99482 | 0.98425 |
| Adj. R-Square | 0.99352 | 0.98088 |

|  | Peak3  ((200) of orthorhombic PCL) | Peak4  ((210) of orthorhombic PCL) |
| --- | --- | --- |
| y0 (arb. units) | 0.07535 ± 0.00302 | 0.0846 ± 0.00208 |
| xc ($Å$^-1^) | 1.67171 ± 8.23311E-5 | 1.71024 ± 5.10849E-4 |
| w ($Å$^-1^) | 0.00856 ± 3.23564E-4 | 0.01873 ± 0.00293 |
| A (arb. units) | 0.00502 ± 1.67849E-4 | 8.52838E-4 ± 1.63611E-4 |
| Reduced Chi-Sqr | 6.39621E-5 | 6.43322E-6 |
| R-Square (COD) | 0.99496 | 0.93393 |
| Adj. R-Square | 0.99402 | 0.91977 |

| (b) hydrated  DPPC (PL) | Peak1  (*d*_20_ of *gel-phase* DPPC) | Peak2  (*d_11/1-1_* of *gel-phase* DPPC) |
| --- | --- | --- |
| y0 (arb. units) | -0.00363 ± 0.00212 | -0.00363 ± 0.00212 |
| xc ($Å$^-1^) | 1.48049 ± 8.68153E-5 | 1.51606 ± 6.5253E-4 |
| w ($Å$^-1^) | 0.00799 ± 2.25218E-4 | 0.1044 ± 0.00383 |
| A (arb. units) | 0.00208 ± 6.4408E-5 | 0.01593 ± 8.61824E-4 |
| Reduced Chi-Sqr | 3.99052E-6 | |
| R-Square (COD) | 0.99824 | |
| Adj. R-Square | 0.99785 | |


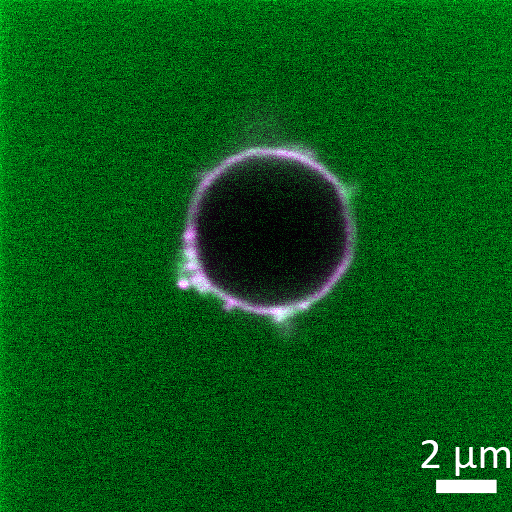

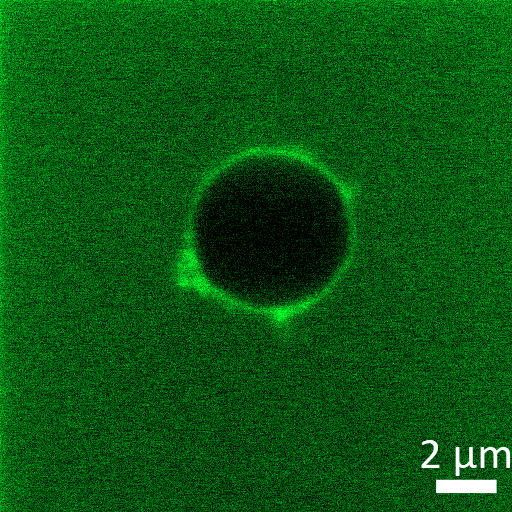

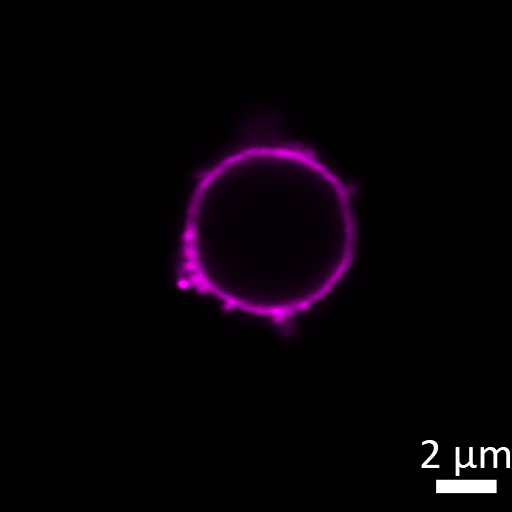

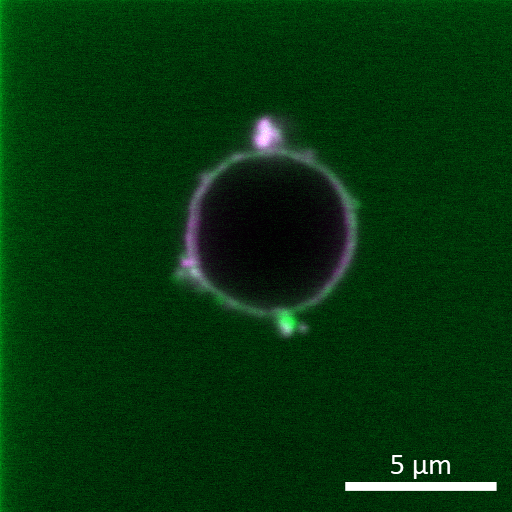

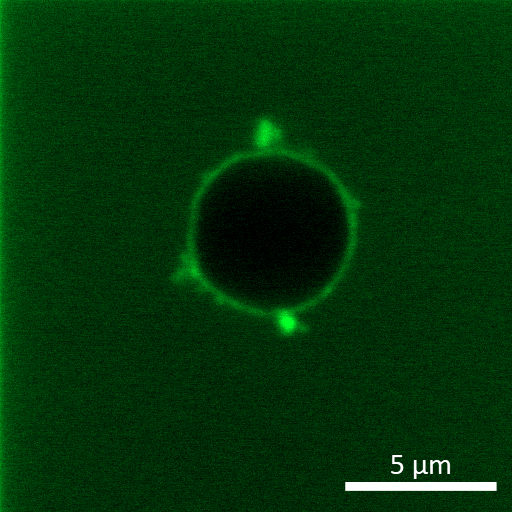

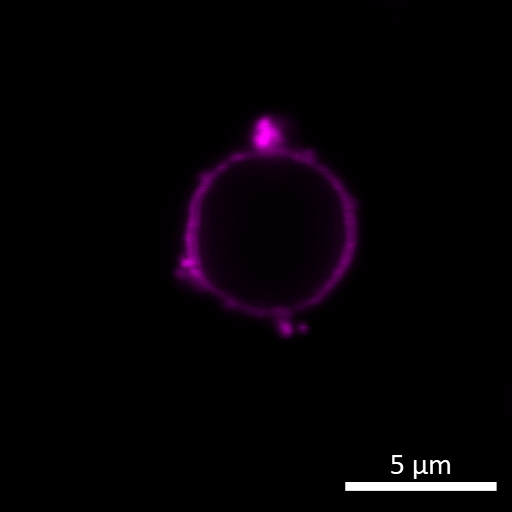


RL-FITC

RL-RhodB

MERGE

(a)

(b)

**Figure S4.** 2D cross-sectional CLSM images of two DPPC - mPEG-*b*-PCL GHUVs in hypertonic media (80 mol% DPPC with respect to mPEG-*b*-PCL) ((a) and (b)). (mPEG-*b*-PCL)-FITC and 16:0 Liss Rhod PE were used to tag polymer domains and lipid domains, respectively. The vesicle encapsulates MilliQ water and the external dispersion medium is introduced with 100 mM glucose solution. RL-FITC fluorescence in green (excitation laser at 488 nm / detection wavelength 400-544 nm) on the *left,* RL-Rhod B fluorescence in magenta (excitation laser at 561 nm / detection wavelength 558-700 nm) in the *middle,* and the merged image of RL-FITC and RL-Rhod B channels on the *right.* All images were obtained on Plan-Apochromat 63×/1.40 Oil DIC M27 objective lens.

**Video S1.** Vertically scanned CLSM video of DPPC - mPEG-*b*-PCL GHUVs in hypertonic media (80 mol% DPPC with respect to mPEG-*b*-PCL); by collecting the 2D cross-sectional CLSM images obtained on different focal planes, or Z-stacking. (mPEG-*b*-PCL)-FITC and 16:0 Liss Rhod PE were used to tag polymer domains and lipid domains, respectively. The vesicle encapsulates MilliQ water and the external dispersion medium is introduced with 100 mM glucose solution. Two separate channels were used; RL-FITC fluorescence in green (excitation laser at 488 nm / detection wavelength 400-544 nm) and RL-Rhod B fluorescence in magenta (excitation laser at 561 nm / detection wavelength 558-700 nm). The video is the merged result of both RL-FITC and RL-Rhod B channels. Z-stack thickness: 0.290 μm. All images were obtained on Plan-Apochromat 63×/1.40 Oil DIC M27 objective lens.


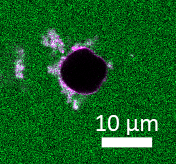

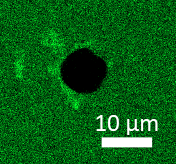


**RL-FITC**

**RL-RhodB**

**MERGE**

(a)

(b)

(c)

**Figure S5.** 2D cross-sectional CLSM images of a DPPC - mPEG-*b*-PCL GHUV with surrounding BCP-PL particulates or debris in hypertonic media (80 mol% DPPC with respect to mPEG-*b*-PCL). (mPEG-*b*-PCL)-FITC and 16:0 Liss Rhod PE were used to tag polymer domains and lipid domains, respectively. The BCP-PL particulates or debris are highlighted by yellow arrows. (a) RL-FITC fluorescence in green (excitation laser at 488 nm / detection wavelength 400-544 nm) (b) RL-Rhod B fluorescence in magenta (excitation laser at 561 nm / detection wavelength 558-700 nm)*,* and (c) the merged image of RL-FITC and RL-Rhod B channels. The vesicle encapsulates MilliQ water and the external dispersion medium is introduced with 100 mM glucose solution. All images were obtained on Plan-Apochromat 63×/1.40 Oil DIC M27 objective lens. One thing to note is the presence of BCP-PL particulates or debris, which is a different morphology to GHUVs.
